# α-Synuclein filaments from transgenic mouse and human synucleinopathy-containing brains are major seed-competent species

**DOI:** 10.1074/jbc.RA119.012179

**Published:** 2020-03-24

**Authors:** Sophie A. Morgan, Isabelle Lavenir, Juan Fan, Masami Masuda-Suzukake, Daniela Passarella, Michael A. DeTure, Dennis W. Dickson, Bernardino Ghetti, Michel Goedert

**Affiliations:** ‡ MRC Laboratory of Molecular Biology, Cambridge CB2 0QH, United Kingdom; § The Mayo Clinic, Jacksonville, Florida 32224; ¶ Department of Pathology and Laboratory Medicine, Indiana University School of Medicine, Indianapolis, Indiana 46202

**Keywords:** alpha-synuclein (α-synuclein), oligomer, neurodegeneration, Parkinson disease, fibril, brain disease, Lewy body, multiple system atrophy, seeded aggregation, synucleinopathy

## Abstract

Assembled α-synuclein in nerve cells and glial cells is the defining pathological feature of neurodegenerative diseases called synucleinopathies. Seeds of α-synuclein can induce the assembly of monomeric protein. Here, we used sucrose gradient centrifugation and transiently transfected HEK 293T cells to identify the species of α-synuclein from the brains of homozygous, symptomatic mice transgenic for human mutant A53T α-synuclein (line M83) that seed aggregation. The most potent fractions contained Sarkosyl-insoluble assemblies enriched in filaments. We also analyzed six cases of idiopathic Parkinson's disease (PD), one case of familial PD, and six cases of multiple system atrophy (MSA) for their ability to induce α-synuclein aggregation. The MSA samples were more potent than those of idiopathic PD in seeding aggregation. We found that following sucrose gradient centrifugation, the most seed-competent fractions from PD and MSA brains are those that contain Sarkosyl-insoluble α-synuclein. The fractions differed between PD and MSA, consistent with the presence of distinct conformers of assembled α-synuclein in these different samples. We conclude that α-synuclein filaments are the main driving force for amplification and propagation of pathology in synucleinopathies.

## Introduction

Assemblies of filamentous α-synuclein define a group of neurodegenerative diseases called synucleinopathies (1). Parkinson's disease (PD),^2^ the most common synucleinopathy, is characterized by the presence of Lewy body and Lewy neurite inclusions in a number of brain regions, including the substantia nigra (2, 3). Lewy pathology also defines Lewy body dementia, which encompasses dementia with Lewy bodies (DLB) and Parkinson's disease dementia (PDD) (2–4). Unlike PD, in Lewy body dementia, α-synuclein pathology is predominant in neocortical and limbic regions, as well as in substantia nigra (5, 6). Multiple system atrophy (MSA) is defined by the presence of abundant filamentous α-synuclein inclusions in oligodendrocytes, also known as glial cytoplasmic inclusions or Papp-Lantos bodies (7–10); smaller numbers of α-synuclein inclusions are present in nerve cells (11, 12). Cases of MSA are classified as MSA-P, with predominant parkinsonism caused by striatonigral degeneration, and MSA-C, with cerebellar ataxia associated with olivopontocerebellar atrophy (13). Autonomic dysfunction is common to both MSA-P and MSA-C.

α-Synuclein is a 140-amino acid protein, which is natively unfolded (1). It interacts with membranes of different lipid composition, and binds preferentially to lipids with high curvature (14, 15). α-Synuclein is believed to facilitate exocytosis of neurotransmitters through dilation of synaptic vesicle pores (16). In synucleinopathies, there is a conversion from monomeric to filamentous α-synuclein. To understand disease pathogenesis, it is essential to work out how α-synuclein assembles into filaments and how these assemblies propagate through the brain.

Six missense mutations in *SNCA,* the α-synuclein gene, cause familial PD (17–23); duplications and triplications of *SNCA* also give rise to PD (24–26). The brains of individuals with these mutations are littered with α-synuclein inclusions that are indistinguishable from those in sporadic PD, indicating that dysfunction or overexpression of α-synuclein is sufficient to cause assembly and neurodegeneration.

α-Synuclein inclusions originate in distinct parts of the brain, from where they appear to spread to anatomically connected regions (27, 28). Based on this, a staging system for PD has been proposed (29). Apparent spread of assembled α-synuclein has also been observed in PD patients who received fetal mesencephalic grafts for therapeutic relief (30, 31). Transfer of assembled α-synuclein has been shown experimentally, both *in vivo* and *in vitro*. Grafts implanted in transgenic mice developed α-synuclein inclusions, demonstrating inter-neuronal spread (32, 33). Cell-based assays have also demonstrated the transfer of α-synuclein between cells (34) and have identified some pathways for internalization (35).

Noncell-autonomous mechanisms have come to explain how toxic amyloid aggregates can spread through prion-like mechanisms (36). Intracerebral injection of brain homogenates has been shown to induce or promote the assembly of α-synuclein (37–39). Injection of filaments assembled from recombinant α-synuclein also induced aggregation and neurodegeneration (40, 41). Moreover, peripheral injections of assembled α-synuclein seeded α-synuclein aggregation in the brains of transgenic mice (42–45). A recent study reported that injection of α-synuclein filaments assembled from recombinant protein into the muscle layers of pylorus and duodenum of WT mice led to the assembly of α-synuclein and neurodegeneration in brain regions affected in PD (46).

The most seed-competent species of assembled α-synuclein are only poorly understood. We therefore used sucrose gradient centrifugation and transiently transfected human embryonic kidney (HEK 293T) cells to identify α-synuclein seeds from the brains of homozygous, symptomatic mice transgenic for human A53T α-synuclein (line M83) (47), and from PD and MSA brains.

## Results

### HEK 293T cell-seeding assay

**Figure 1.**
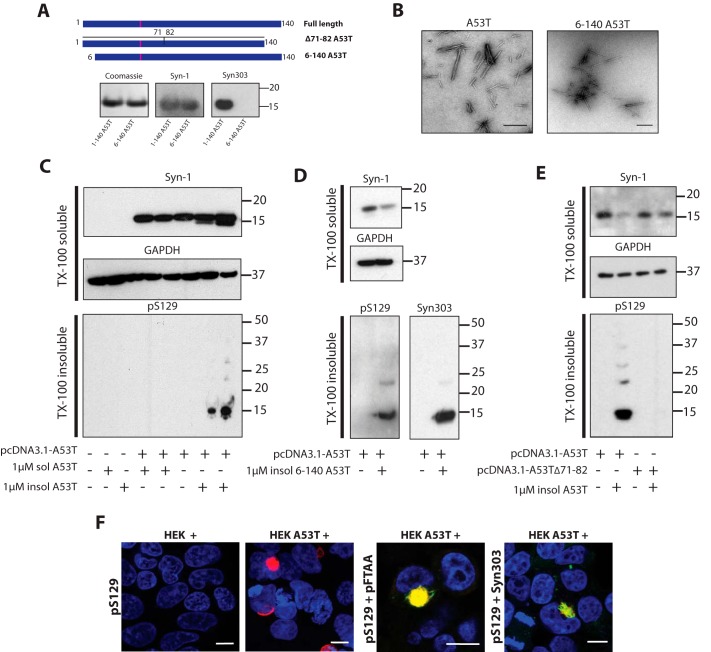
**Seeding assay for A53T α-synuclein in HEK 293T cells.** *A,* full-length 1–140 and N terminally truncated 6–140 A53T α-synuclein in pRK172 were expressed in *E. coli* BL21 (DE3) cells. Following purification (*Coomassie*), immunoblotting showed that Syn-1 labeled both full-length and truncated α-synuclein, whereas Syn303 labeled full-length, but not truncated, α-synuclein. After transient transfection, full-length and Δ71–82 A53T α-synuclein were expressed in HEK 293T cells. *B,* electron micrographs of assembled full-length and truncated A53T α-synuclein. *Scale bar*, 500 nm. *C,* Western blot analysis of untransfected and A53T α-synuclein–transfected HEK 293T cells seeded with unassembled and assembled full-length A53T α-synuclein. Cell lysates were separated into Triton X-100–soluble and -insoluble fractions by ultracentrifugation at 100,000 × *g* for 1 h at 4 °C. Blots of anti-α-synuclein antibody Syn-1 and anti-GAPDH were processed for the Triton X-100–soluble fractions and anti-α-synuclein antibody pS129 was used for Triton X-100–insoluble fractions. Twenty μg of Triton X-100–soluble and 50 μg of Triton X-100–insoluble proteins were run on 4–12% BisTris SDS-PAGE. *D,* Western blot analysis of full-length A53T α-synuclein expressing HEK 293T cells seeded with N terminally truncated A53T α-synuclein aggregates. Triton X-100–soluble fractions were blotted with Syn-1 and anti-GAPDH. Triton X100–insoluble fractions were blotted with pS129 and Syn303. *E,* Western blot analysis of full-length and Δ71–82 A53T α-synuclein expressing HEK 293T cells seeded with 1 μm full-length α-synuclein aggregates. Triton X-100–soluble fractions were blotted with Syn-1 and anti-GAPDH antibodies. Triton X-100–insoluble fractions were blotted with anti-pS129 antibody. *F,* staining of untransfected (HEK+) and full-length A53T α-synuclein–transfected (HEK A53T+) cells seeded with 1 μm aggregated N terminally truncated 6–140 A53T α-synuclein with pS129 (*red*), Syn303 (*green*), and pFTAA (*green*). Nuclei were visualized with DAPI (*blue*). *Scale bar*, 10 μm.

Recombinant full-length (1–140) and N terminally truncated (6–140) human A53T α-synucleins were expressed, purified, and assembled into filaments (Fig. 1, *A* and *B*). They were then used as seeds following transient expression of full-length A53T α-synuclein in HEK 293T cells. Addition of seeds induced the formation of Triton X-100–insoluble α-synuclein that was positive with an antibody specific for phosphorylation at serine 129 (pS129) (Fig. 1*C*). Assembled α-synuclein is phosphorylated at Ser-129 *in vivo* (48, 49). Nonassembled α-synuclein did not induce aggregation (Fig. 1*C*). To establish that insoluble α-synuclein was different from the seeds, assembled (6–140) A53T α-synuclein was used. Antibody Syn303 is specific for amino acids (1–5) of α-synuclein (50). Seeds of 6–140 A53T α-synuclein induced the formation of Triton X-100–insoluble α-synuclein that was positive with antibodies pS129 and Syn303 (Fig. 1*D*), showing that the assemblies were made of full-length α-synuclein. When Δ71–82 A53T α-synuclein, which is unable to assemble (51), was expressed, no seeded aggregation was observed (Fig. 1*E*). Moreover, immunohistochemistry showed perinuclear and skein-like inclusions, which were positive for antibody pS129 and the luminescent-conjugated oligothiophene pFTAA, in both transfected and seeded and cells (Fig. 1*F*). pFTAA stains the pS129–positive α-synuclein inclusions of PD and MSA (52).

### Characterization of α-synuclein from the brains of mice transgenic for human A53T α-synuclein (line M83)

**Figure 2.**
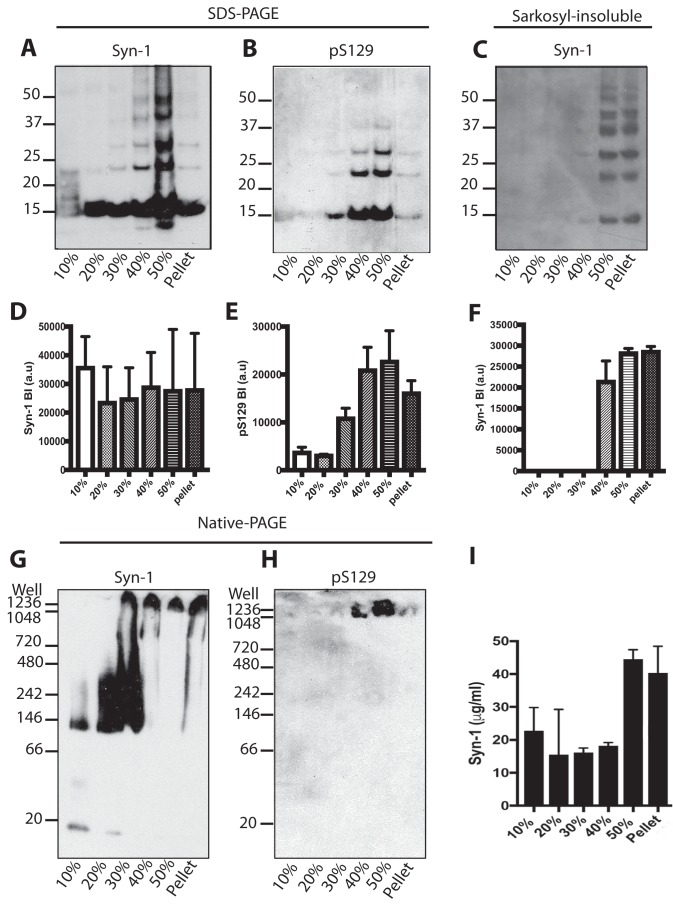
**Characterization of α-synuclein from the brains of homozygous M83 mice following sucrose gradient centrifugation.** *A* and *B,* Western blot analysis (anti-α-synuclein antibodies Syn-1 and pS129) following SDS-PAGE of brain lysates fractionated by sucrose gradient centrifugation. A representative blot is shown. *C,* Western blot analysis (antibody Syn-1) of Sarkosyl-insoluble brain lysates. *D–F,* densitometric analysis of fractionated brain lysates and Sarkosyl-insoluble lysates. The results are the mean ± S.D. (*n* = 3). *G* and *H,* Western blot analysis (anti-α-synuclein antibodies Syn-1 and pS129) following native-PAGE of brain lysates fractionated by sucrose gradient centrifugation. *I*, to quantify the amount of α-synuclein in the sucrose gradient fractions, dot-blot analysis was performed with α-synuclein protein standards. The results are expressed as the mean ± S.D. (*n* = 3).

We used sucrose gradient centrifugation to separate α-synuclein assemblies from the brains of homozygous, symptomatic mice transgenic for human A53T α-synuclein (line M83). Sucrose densities were: 50, 40, 30, 20, and 10% in PBS. Brain lysates were spun at 281,000 × *g* for 4 h at 20 °C. Fractions were analyzed by Western blotting with anti-α-synuclein antibodies Syn-1 and pS129 to detect total and phosphorylated α-synuclein, respectively. By SDS-PAGE, all fractions contained monomeric α-synuclein that ran as a 15-kDa band (Fig. 2, *A*, *C*, *D*, and *F*). It is well-established that some higher molecular mass bands, such as those at 22 and 29 kDa, correspond to ubiquitinated α-synuclein (49, 53–55) Assembled α-synuclein, which was pS129–positive, was present in the 30, 40, 50%, and pellet fractions, with highest levels in the 40 and 50% fractions (Fig. 2, *B* and *E*). Sarkosyl-insoluble α-synuclein was detected in the 40, 50%, and pellet fractions (Fig. 2, *C* and *F*). Native-PAGE Western blot analysis with Syn-1 revealed monomeric α-synuclein in the 10% sucrose fraction, which ran at ∼15 kDa and a higher molecular mass species that ran at 140 kDa (Fig. 2*G*). The 20% fraction contained predominantly higher molecular mass multimeric α-synuclein, which ranged from 140 to 480 kDa. The same was true of the 30% fraction, which contained in addition multimeric α-synuclein between the well and 480 kDa. Moreover, this fraction also contained α-synuclein that did not enter the gel. Material that did not enter the gel was also present in the 40, 50%, and pellet fractions. Antibody pS129 only recognized α-synuclein species that were trapped in the wells (Fig. 2*I*). The amount of α-synuclein in each fraction was determined by dot-blotting with Syn-1 (Fig. 2*F*). In contrast to homozygous M83 mice, heterozygous mice did not develop motor dysfunction. Sucrose gradient fractionated brain extracts from heterozygous M83 mice were unable to seed aggregation of human A53T α-synuclein in transfected HEK 293T cells (Fig. S1).

### Immunoelectron microscopy

**Figure 3.**
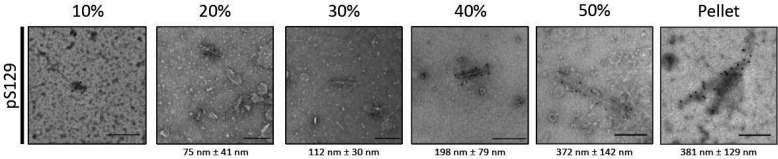
**Immunoelectron microscopy of brain lysates from homozygous M83 mice following sucrose gradient fractionation.** *A,* immunoelectron microscopy of fractionated brain lysates from homozygous, symptomatic mice transgenic for human A53T α-synuclein (line M83) with anti-α-synuclein pS129 antibody. The measured filament lengths (20, 30, 40, 50%, and pellet) are shown (*n* = 15). *Scale bar*, 200 nm.

Immunoelectron microscopy using an antibody specific for α-synuclein phosphorylated at Ser-129 was used to study the presence of α-synuclein assemblies in sucrose gradient fractions from the brains of mice transgenic for human A53T α-synuclein (Fig. 3). In the 10% fraction, we did not detect any pS129–positive α-synuclein assemblies. The 20% fraction contained pS129–positive filaments of 75 ± 41 nm. Filaments in the 30% fraction were 112 ± 30 nm, 198 ± 79 nm in the 40% fraction, and 372 ± 142 nm in the 50% fraction (Fig. 3).

### Seeded aggregation of α-synuclein with sucrose gradient-fractionated brain lysates from mice transgenic for human A53T α-synuclein

**Figure 4.**
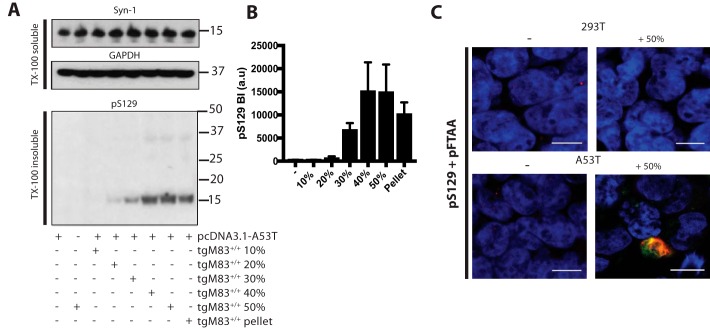
**Seeding potency of sucrose gradient fractionated brain lysates from homozygous M83 mice.** *A,* Western blot analysis of untransfected and full-length human A53T α-synuclein–transfected HEK 293T cells seeded with sucrose gradient-fractionated homozygous M83 brain lysates. Cells were incubated with sucrose gradient fractions for 72 h and harvested for Western blot analysis. Cell lysates were separated into Triton X-100–soluble and –insoluble fractions by ultracentrifugation at 100,000 × *g* for 1 h at 4 °C. Twenty μg of protein of the supernatant was loaded onto 4–12% BisTris SDS-PAGE for Triton X-100–soluble fractions and 50 μg of protein from the pellet for Triton X-100–insoluble fractions. A representative Western blotting is shown. *B,* densitometric analysis of Triton X-100–insoluble pS129 in seeded cells. The results are expressed as the mean ± S.D. (*n* = 3). *C,* staining of untransfected (293T) and full-length A53T α-synuclein–transfected (A53T) cells unseeded (−) and seeded with 50% sucrose fraction using antibody pS129 (*red*) and LCO pFTAA (*green*). Nuclei were visualized with DAPI (*blue*). *Scale bar*, 10 μm.

The ability of fractionated assemblies to seed aggregation was investigated in the HEK 293T cell-seeding assay. Dot-blot analysis was used to quantify α-synuclein levels. One μg of α-synuclein from each fraction was then added to HEK 293T cells expressing human A53T α-synuclein and incubated for 72 h. The 30, 40, and 50% sucrose fractions, as well as the pellet, induced seeded aggregation (Fig. 4, *A* and *B*). 10 and 20% fractions were inactive. The 40 and 50% fractions were the most potent, followed by pellet and 30% fractions. Immunohistochemistry with anti-α-synuclein antibody pS129 and staining with pFTAA confirmed seeded aggregation (Fig. 4*C*). When HEK 293T cells that did not express α-synuclein were seeded with the 50% sucrose fraction, no staining was observed with the pS129 antibody.

### α-Synuclein assemblies from Parkinson's disease and multiple system atrophy brains seed aggregation

**Figure 5.**
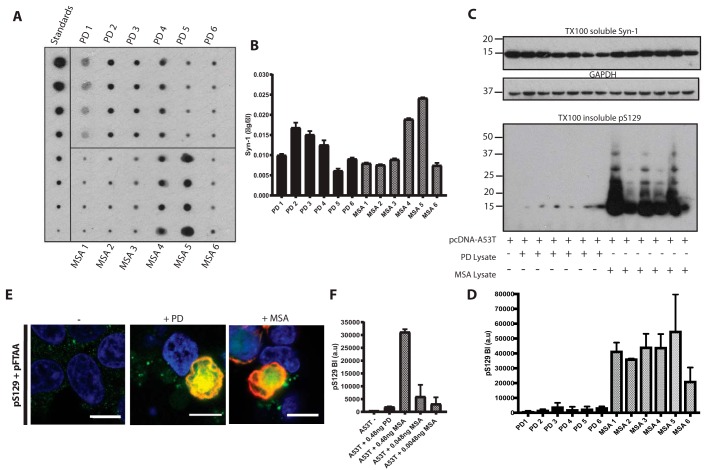
**Parkinson's disease and multiple system atrophy brain lysates induce α-synuclein aggregation in transfected HEK 293T cells.** *A* and *B,* to quantify α-synuclein in six idiopathic PD (PD1–6) and six MSA (MSA1–6) brain lysates, we used dot blotting with anti-α-synuclein antibody Syn-1. Recombinant α-synuclein protein standards were blotted alongside diluted brain lysates (*n* = 3). *C,* Western blot analysis of human A53T full-length α-synuclein–transfected cells seeded with 1 ng of α-synuclein from each of the PD and MSA brain lysates. Triton X-100–soluble fractions were blotted with Syn-1 and anti-GAPDH antibodies, and Triton X-100–insoluble fractions were blotted with anti-α-synuclein pS129 antibody. *D,* densitometric analysis of Triton X-100–insoluble pS129 band intensities from seeded HEK 293T cells. *E,* staining of human A53T α-synuclein–transfected cells unseeded (−) and seeded with PD (+*PD*) and MSA (+*MSA*) brain lysates stained for anti-α-synuclein antibody pS129 (*red*) and pFTAA (*green*). Nuclei were visualized with DAPI (*blue*). *Scale bar*, 10 μm. *F,* densitometric analysis of human A53T α-synuclein–transfected cells seeded with 0.48 ng of PD lysate and serially diluted MSA lysate. The results are expressed as the mean ± S.D. (*n* = 3).

α-Synuclein levels were measured by dot blotting of substantia nigra from six cases of neuropathologically confirmed idiopathic PD and cerebellum from six cases of neuropathologically confirmed MSA-C using recombinant α-synuclein standards (Fig. 5, *A* and *B*). One ng of α-synuclein from each brain lysate was added to HEK 293T cells expressing human A53T α-synuclein and incubated for 72 h. Western blot analysis showed that MSA brain lysates induced α-synuclein assembly more robustly than PD lysates (Fig. 5, *C*, *D*, and *F*). Staining confirmed the presence of α-synuclein assemblies in seeded cells, which were positive using antibody pS129 and pFTAA (Fig. 5*E*). In some experiments, superior temporal gyrus from a case of neuropathologically confirmed PD and diffuse DLB with a heterozygous duplication of *SNCA* was used (56).

### Characterization of α-synuclein from Parkinson's disease brains and identification of seed-competent species

**Figure 6.**
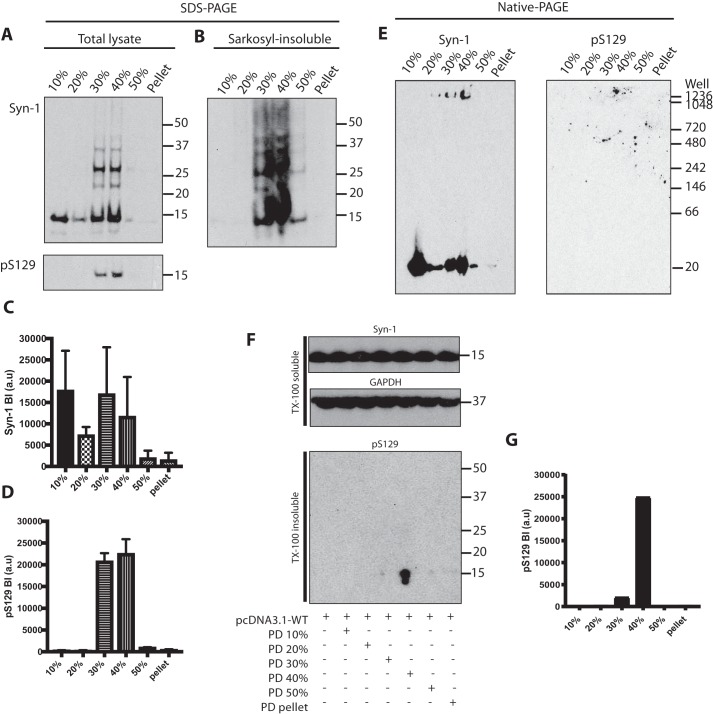
**Characterization of α-synuclein from Parkinson's disease brains following sucrose gradient centrifugation and identification of seed-competent species.** *A,* Western blot analysis (anti–α-synuclein antibodies Syn-1 and pS129) following SDS-PAGE of brain lysates fractionated by sucrose gradient centrifugation. A representative blot using PD2 is shown. *B,* Western blot analysis (antibody Syn-1) of Sarkosyl-insoluble brain lysates. *C* and *D,* densitometric analysis of fractionated brain lysates. The results are expressed as the mean ± S.D. (*n* = 3). *E,* Western blot analysis (anti-α-synuclein antibodies Syn-1 and pS129) following native-PAGE of brain lysates fractionated by sucrose gradient centrifugation. *F,* Western blot analysis following SDS-PAGE of HEK 293T cells expressing human WT α-synuclein seeded with fractionated lysates of the superior temporal gyrus from an *SNCA* duplication case. Triton X-100–soluble cell lysates were blotted with Syn-1 and anti-GAPDH antibodies. Triton X-100 insoluble fractions were blotted with pS129. *G,* densitometric analysis of Triton X-100–insoluble pS129 bands of seeded cells.

Brain lysates of substantia nigra from two cases of idiopathic PD (PD2 and PD3) and the case of familial PD were fractionated by sucrose gradient centrifugation. Western blot analysis with Syn-1 showed monomeric α-synuclein in all fractions and higher molecular weight α-synuclein in the 30 and 40% fractions (Fig. 6, *A–D*). Anti-pS129 antibody was only positive with material in the 30 and 40% fractions (Fig. 6*A*). These fractions contained Sarkosyl-insoluble α-synuclein, as did the 50% fraction (Fig. 6*B*). Nondenaturing gel electrophoresis showed the presence of high-molecular weight assemblies of α-synuclein that did not migrate into the gel in the 30 and 40% fractions, and confirmed the presence of monomeric α-synuclein in most fractions (Fig. 6*E*). Brain lysates from PD2 and PD3, as well as from the case of familial PD, were tested in the cell-seeding assay. One ng of α-synuclein from each fraction was added to HEK 293T cells expressing WT α-synuclein. The case of familial PD (Fig. 6, *F* and *G*), but not PD2 or PD3, seeded aggregation. Only the 40% sucrose gradient fraction seeded robustly. Occasionally, the 30% fraction also seeded.

### Characterization of α-synuclein from multiple system atrophy brains and identification of seed-competent species

**Figure 7.**
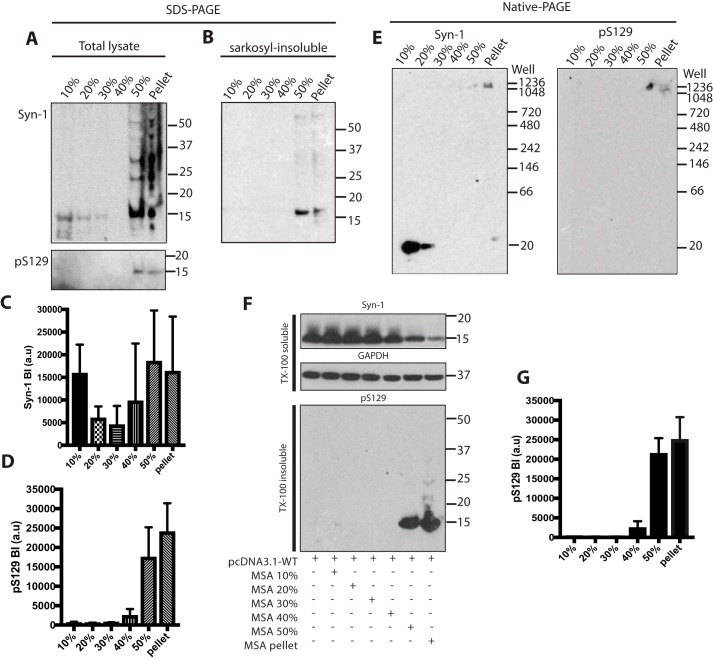
**Characterization of α-synuclein from multiple system atrophy brains following sucrose gradient centrifugation and identification of seed-competent species.** *A,* Western blot analysis (anti-α-synuclein antibodies Syn-1 and pS129) following SDS-PAGE of brain lysates fractionated by sucrose gradient centrifugation. A representative blot using MSA5 is shown. *B,* Western blot analysis (antibody Syn-1) of Sarkosyl-insoluble brain lysates. *C* and *D,* densitometric analysis of fractionated brain lysates. *E,* Western blot analysis (anti-α-synuclein antibodies Syn-1 and pS129) following native-PAGE of brain lysates fractionated by sucrose gradient centrifugation. *F,* Western blot analysis following SDS-PAGE of HEK cells expressing human WT α-synuclein seeded with fractionated lysates. Triton X-100–soluble cell lysates were blotted with Syn-1 and anti-GAPDH antibodies. Triton X-100–insoluble fractions were blotted with pS129. *G,* densitometric analysis of Triton X-100–insoluble pS129 bands of seeded cells. Densitometric results are expressed as the mean ± S.D. (*n* = 3).

Brain lysates of cerebellum from cases MSA1, MSA4, and MSA5 were fractionated by sucrose gradient centrifugation. Western blot analysis with Syn-1 showed monomeric α-synuclein in all fractions and higher molecular weight α-synuclein in the 50% and pellet fractions (Fig. 7, *A–D*). Anti-pS129 antibody was only positive with material in the 50% and pellet fractions (Fig. 7*A*). These fractions contained Sarkosyl-insoluble α-synuclein (Fig. 7*B*). Nondenaturing gel electrophoresis showed the presence of high-molecular weight assemblies of α-synuclein that did not migrate into the gel in the 50% and pellet fractions, and confirmed the presence of monomeric α-synuclein in the less dense fractions (Fig. 7*E*). One ng of α-synuclein from each fraction was added to HEK 293T cells expressing WT α-synuclein. Only the 50% and pellet fractions seeded robustly. Occasionally the 40% fraction also seeded (Fig. 7, *F* and *G*). All three cases of MSA gave similar results.

## Discussion

Assembled α-synuclein can spread between anatomically connected neurons and seed the aggregation of soluble α-synuclein into filaments (57). α-Synuclein species responsible for seeding are not completely known. We therefore used sucrose gradient centrifugation (58) to analyze brain lysates from symptomatic mice transgenic for human mutant A53T α-synuclein (line M83) (47); such lysates support α-synuclein propagation (37, 38). Highly aggregated forms pellet more rapidly than amorphous aggregates, small oligomers, and monomeric α-synuclein.

Analysis of brain lysates by SDS-PAGE following sucrose gradient centrifugation revealed the presence of monomeric and multimeric α-synuclein. Syn-1 identified monomeric α-synuclein in all fractions. Ubiquitinated α-synuclein was mostly present in the 40 and 50% fractions. The latter were also labeled predominantly by an antibody specific for α-synuclein phosphorylated at Ser-129. The 40 and 50% sucrose gradient fractions were the most potent at seeding aggregation of α-synuclein in the HEK 293T cell assay. The pellet fraction was slightly less effective, followed by the 30% sucrose fraction. The 10 and 20% fractions were devoid of significant activity. Sarkosyl-insoluble α-synuclein was present in the 40, 50%, and pellet fractions. Because these fractions are enriched in filaments and because filaments have been shown to seed aggregation (38, 59), it appears likely that α-synuclein filaments of 198–381 nm were the most seed-competent species. Previous work using α-synuclein assembled from recombinant protein and untransfected SH-SY5Y cells also showed that short filaments were the most seed-potent (60). Moreover, sonicated filaments assembled from recombinant human α-synuclein were more potent at seeding assembly than nonsonicated filaments (61).

By native PAGE, several types of α-synuclein assemblies were observed upon fractionation of brain lysates from symptomatic M83 mice. Syn-1–positive α-synuclein resolved as a ladder ranging from 140 to 1,200 kDa in the 10–30% fractions. The 30% fraction also contained high-molecular weight α-synuclein (>1,240 kDa). The latter, which did not enter the gel, was the major species in the 40, 50%, and pellet fractions. The pS129 α-synuclein antibody only labeled material that did not enter the gel.

These findings are reminiscent of those obtained for tau protein from the brains of symptomatic mice transgenic for human mutant P301S tau following sucrose gradient fractionation and seeding in HEK 293T cells (62). Short filaments were the major species of seed-competent tau. It thus appears that filamentous tau and filamentous α-synuclein are the molecular species with the most seeding activity. Monomeric proteins and small oligomers were inactive. These findings are in contrast to those reporting that some species of tau monomers were able to seed aggregation of human mutant, truncated tau in a biosensor cell line (63). Although human A53T α-synuclein was transiently expressed in the present study, previous work (62) used HEK 293T cells stably expressing human 0N4Rtau. It remains to be seen if stable expression of A53T α-synuclein will result in less variability.

Different conformers of assembled α-synuclein may be characteristic of PD and MSA (64–69). Thus, previous studies have demonstrated α-synuclein seeding activity in MSA, but not PD, brains (65, 66). We found that α-synuclein seeds from the cerebellum of MSA patients seeded aggregation more potently than seeds from the substantia nigra of PD patients. However, in contrast to the previous work (65, 66), we found that PD brain extracts were also seed-competent in HEK 293T cells expressing human mutant A53T α-synuclein. When expressed relative to a constant amount of α-synuclein, MSA extracts were ∼100 times more potent at seeding aggregation than PD extracts. It is possible that MSA extracts contained more seed-competent α-synuclein than PD extracts, but such differences are unlikely to have been as high as 100-fold. Recently, a biosensor cell line stably expressing A53T α-synuclein-CFP/YFP fusion proteins was used to detect α-synuclein seeds from PD and MSA brains (70).

When fractionating substantia nigra extracts from PD and cerebellar extracts from MSA, the levels of pS129 α-synuclein and Sarkosyl-insoluble protein were highest in the 30 and 40% fractions for PD, and in the 50% and pellet fractions for MSA. Because sucrose gradient centrifugation separates proteins according to size and density, these differences are consistent with the existence of distinct conformers of assembled α-synuclein. Following sucrose gradient centrifugation, the 50% and pellet fractions from the MSA cases tested readily seeded aggregation of WT α-synuclein. By contrast, the 30 and 40% fractions of only one case of PD (familial disease caused by duplication of *SNCA*) seeded aggregation. Future experiments will use additional cases of idiopathic PD.

The existence of distinct conformers of assembled α-synuclein is also supported by the morphological differences between isolated, negatively stained α-synuclein filaments from PD and DLB brains when compared with MSA brains (9, 72, 73). To establish the existence of distinct filament conformers unambiguously and to understand their differences, high-resolution structures of α-synuclein filaments from human brain are required.

## Experimental procedures

### Cloning

α-Synuclein cDNAs were amplified from pRK172 vectors by polymerase chain reaction (PCR) with PFU Turbo polymerase. Thermocycles were performed using a Veriti 96-well-thermal cycler (Applied Biosciences). The primers used for constructs to be cloned into pcDNA3.1 mammalian expression vector (Invitrogen) contained a Kozak consensus sequence (ACCACCAUGG) and restriction endonuclease sites (5′) HindIII and (3′) EcoRV. Primer sequences for WT and mutant full-length α-synuclein were as follows: forward primer: 5′-AAGCTTCCACCATGGATGTATTCATG-3′ and reverse primer: 5′GATATCTTAGGCTTCAGGTTC3′. α-Synuclein constructs 6–140 and Δ71–82 were generated using site-directed mutagenesis (Aligent QuikChange Lightening Kit) to remove the relevant amino acids in the human A53T α-synuclein pRK172 plasmid. The designed primer sequences were 5′-CATATGAAAGGACTTTCAAAGGCC-3′ and 5′-AAAAGCTTTTAGGCTTCAGGTTC-3′ for 6–140 A53T α-synuclein and 5′-ACAAATGTTGGAGGAGAGGGAGCAGGGA-3′ and 5′-TCCCTGCTCCCTCTCCTCCAACATTTGT-3′ for Δ71–82 A53T α-synuclein.

### Recombinant α-synuclein filaments

Full-length (1–140) and truncated (6–140) human A53T α-synucleins were expressed using pRK172 vectors in *Escherichia coli* BL21 (DE3) cells and purified using modifications of a published protocol (74). Briefly, anion-exchange chromatography using a HiTrap Q HP column (GE Life Sciences) was followed by size exclusion chromatography on a HiLoad 16/600 Superdex 200 column (GE Life Sciences). α-Synuclein was concentrated to 400 μm in 3K cut-off ultrafiltration spin columns (Vivaspin), dialyzed against phosphate-buffered saline (PBS) overnight at 4 °C, and assembled using rotational shaking (450 rpm) at 37 °C for 5 days. Assembled protein was spun at 100,000 × *g* for 1 h at 4 °C and the pellets resuspended in PBS at 400 μm, followed by a 30-s sonication (Misonix XL).

### Brain lysates

Transgenic mouse line M83, which expresses human A53T α-synuclein under the control of the mouse prion protein promoter (47), was purchased from the Jackson Laboratory (stock number 004479). Symptomatic, homozygous M83 mice aged 14 (female), 16 (male), and 24 (female) months were used. Heterozygous M83 mice used were aged 6 (female), 10 (male), and 14 (male) months. Experiments were carried out in compliance with the UK Animals (Scientific Procedures) Act of 1986 and were approved by the local Animal Welfare and Ethical Review Board. Mice were euthanized by cervical dislocation, followed by exsanguination. Whole brains were dissected, frozen on dry ice, and stored at −80 °C. They were homogenized at 10% (w/v) in sterile PBS plus Complete protease inhibitor mixture (Roche Applied Science) using a Polytron (PT 2100 Kinematica AG). Homogenates were spun at 5,000 × *g* for 5 min at 4 °C. Supernatants were stored in aliquots at −80 °C until use. α-Synuclein levels were measured by dot blotting. Fresh-frozen substantia nigra from six cases of neuropathologically confirmed idiopathic PD (1 woman (PD1), age at death, 74 years; and 5 men (PD2-PD6), ages at death, 68, 83, 74, 79, and 75 years), superior temporal gyrus from a man with neuropathologically confirmed familial PD and diffuse DLB (*SNCA* duplication, age at death, 63 years) and cerebellum from six neuropathologically confirmed cases of MSA-C (2 men (MSA2, MSA5), ages at death, 77 and 52 years; and 4 women (MSA1, MSA3, MSA4, MSA6), ages at death, 81, 65, 71, and 83 years) were homogenized in sterile PBS at 200 mg/ml using a Polytron (PT 2500 E, Kinematica AG). Samples were kept on ice and centrifuged at 5,000 rpm at 4 °C for 5 min. Supernatants were stored in aliquots at −80 °C.

### Sucrose gradient centrifugation

Ten, 20, 30, 40, and 50% sucrose fractions were prepared in PBS and layered onto 5-ml centrifuge tubes (BeckmanCoulter Ultra-Clear). Volumes were: 0.5 ml at 50%, 0.5 ml at 40%, 0.5 ml at 30%, 0.5 ml at 20%, and 1 ml at 10% sucrose, layered in decreasing density. One ml of brain lysate (15–20 mg of protein) was added on top of the gradient. The tubes were spun at 250,000 × *g* in a Beckman Ti 60 swing-out rotor for 4 h at 20 °C (58). Fractions were collected and stored at −80 °C. Pellets were resuspended in 0.5 ml of PBS.

### Sarkosyl extraction

One hundred μl of the sucrose gradient fractions were incubated with 1% Sarkosyl for 1 h at 22 °C on a flat rotational shaker (700 rpm). Samples were centrifuged at 100,000 × *g* for 1 h at 4 °C and pellets were resuspended in 50 mm Tris-HCl, pH 7.4.

### Dot blotting

Recombinant α-synuclein proteins of known concentration and brain lysates diluted 1:1,000 were vacuum pulled onto a polyvinylidene fluoride (PVDF) membrane (0.22 μm) using a minifold I spot-blot system (GE Healthcare). Membranes were blocked in 5% milk for 1 h and incubated with anti-α-synuclein antibody Syn-1 (1:1,000, Transduction Laboratories) for 1 h at room temperature, washed three times in PBS-Tween, incubated in horseradish peroxidase secondary antibody in 5% milk for 1 h, and washed again. Chemiluminescence was used to quantify the signal. Serial dilutions (0.5–3.5 ng) of recombinant α-synuclein were used as standard. The linear parts of the standard curves were used to determine the concentrations of α-synuclein. Syn-1 recognizes residues 91–99 of human α-synuclein (71).

### EM

Recombinant α-synuclein aggregates and sucrose gradient fractions of M83 brain lysates were placed onto 400-meshed Formvar/carbon film-coated, glow-discharged copper grids (Sigma) for 3 min, blocked with distilled water + 0.1% gelatin for 10 min, and incubated with anti-α-synuclein antibody pS129 (1:50, ab51253, Abcam) for 3 h in a humidity chamber. Grids were washed in distilled water and incubated with 10-nm gold-conjugated anti-rabbit secondary antibody (Sigma, 1:25) for 1 h, washed, and stained with 0.1% uranyl acetate. Images were taken on a Philips Spirit transmission microscope and measurements made in Fiji (ImageJ).

### HEK 293T cell-seeding assay

HEK 293T cells were transiently transfected with 1 μg of WT human α-synuclein, A53T α-synuclein, or Δ71–82 A53T α-synuclein cDNAs in pcDNA3.1 using Lipofectamine 2000 (Invitrogen) and 6-well-plates. Following overnight incubation, the medium (DMEM + GlutaMAX^TM^ with 10% fetal calf serum) was replaced. Cells were then incubated with sucrose gradient fractions. After 24 h, fresh medium was added and the cells were incubated for a further 48 h. They were then harvested, lysed by incubation in Tris-HCl, pH 7.4, + 1% Triton X-100 for 10 min on ice, and homogenized with a cell sonicator (Misonix, Microson Ultrasonic Cell Disruptor). Lysates were separated into soluble and insoluble fractions by centrifugation at 100,000 × *g* for 30 min at 4 °C.

### Gel electrophoresis and Western blotting

Samples were boiled in buffer containing 2-mercaptoethanol, loaded onto Novex 4–12% BisTris gels (Life Technologies), and run at 200 V for 40 min. Proteins were transferred onto PVDF membranes in a semi-dry transfer tank (Bio-Rad), blocked with 5% milk, and immunoblotted with Syn-1 (1:1,000), Syn303 (1:1,000, BioLegend), or pS129 (1:1,000). After incubation with horseradish peroxidase secondary antibody (1:5,000), bands were visualized using chemiluminescence. For native-PAGE, samples were added to buffer (Invitrogen), loaded onto 4–16% Native-PAGE BisTris gels, and run for 2 h at 150 V. Proteins were transferred onto PVDF membranes by semi-dry transfer.

### Immunofluorescence

Cells were seeded and plated onto poly-l-lysine–coated coverslips (BD Biosciences), fixed with 4% paraformaldehyde for 20 min at 37 °C, and permeabilized with 0.1% Triton X-100, 3% BSA in PBS for 1 h. Primary antibodies were incubated overnight at 4 °C, three PBS washes preceded and followed incubation for 1 h with fluorescent secondary antibodies (1:1,000). Stained cells were mounted in Prolong Gold with DAPI and confocal images were taken on a Zeiss710 microscope. Primary antibodies were: pS129 (ab51253, Abcam) at 1:2,000 and Syn303 (BioLegend) at 1:1,000. pFTAA (0.6 μm in PBS) was added to cells for 3 h at room temperature after primary antibody addition.

### Data availability

All data presented are contained within the manuscript.

## Author contributions

S. A. M. and M. G. conceptualization; S. A. M. data curation; S. A. M. formal analysis; S. A. M. writing-original draft; S. A. M. and M. G. writing-review and editing; I. L., M. A. D., D. W. D., and B. G. resources; J. F., M. M.-S., and D. P. methodology.

## Supplementary Material

Supporting Information

## Footnotes

2 The abbreviations used are:

PD: Parkinson's disease
DLB: dementia with Lewy bodies
PDD: Parkinson's disease dementia
MSA: Multiple system atrophy
HEK: human embryonic kidney
PVDF: polyvinylidene fluoride
BisTris: 2-[bis(2-hydroxyethyl)amino]-2-(hydroxymethyl)propane-1,3-diol
GAPDH: glyceraldehyde-3-phosphate dehydrogenase
DAPI: 4′,6-diamidino-2-phenylindole.
