# Supplementary material for: α-Synuclein filaments from transgenic mouse and human synucleinopathy-containing brains are major seed-competent species
Source: J Biol Chem. 2020 Mar 24;295(19):6652–64. doi: 10.1074/jbc.RA119.012179 (PMC7212628; doi:10.1074/jbc.RA119.012179)
Supplement: Supporting Information [file supp_295_19_6652__index.html]

α-Synuclein filaments from transgenic mouse and human synucleinopathy-containing brains are major seed-competent species — α-Synuclein filaments are the major seed-competent species — α-Synuclein filaments from transgenic mouse and human synucleinopathy-containing brains are major seed-competent species — α-Synuclein filaments are the major seed-competent species — Supporting Information 

# α-Synuclein filaments from transgenic mouse and human synucleinopathy-containing brains are major seed-competent species

## Supporting Information

- Supporting Information (to be published online) - Supplementary Figure 1
