# Supplementary material for: α-Synuclein filaments from transgenic mouse and human synucleinopathy-containing brains are major seed-competent species
Source: J Biol Chem. 2020 Mar 24;295(19):6652–64. doi: 10.1074/jbc.RA119.012179 (PMC7212628; doi:10.1074/jbc.RA119.012179)
Supplement: Supporting Information [file supp_RA119.012179_157528_2_supp_486897_q68c5f.docx]

**
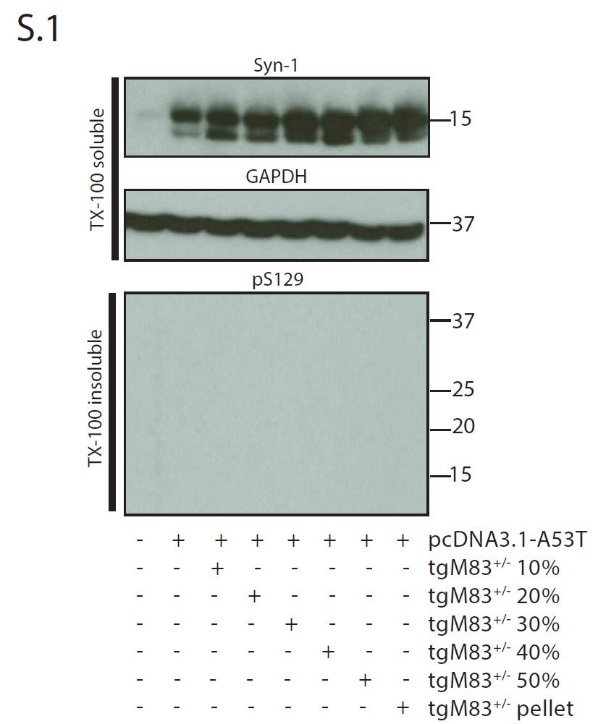
**

**Supplementary Figure 1: Seeding potency of sucrose gradient fractionated brain lysates from heterozygous M83 mice.
A.** Western blot analysis of untransfected and full-length A53T α-synuclein transfected HEK 293T cells seeded with sucrose gradient fractionated heterozygous M83 brain lysates. Cell were incubated with the sucrose gradient fractions for 72 h and harvested for Western blot analysis. Cell lysates were separated into TritonX-100 soluble and insoluble fractions by ultracentrifugation at 100,000 x g for 1 h at 4°C. Twenty μg protein of the supernatant was loaded onto 4-12% BisTris SDS-PAGE for TritonX-100 soluble fractions and 50 μg protein from the pellet for Triton-X100 insoluble fractions.
